# Perfusion in hand arthritis on dynamic contrast-enhanced computed tomography: a randomized prospective study using MRI as a standard of reference

**DOI:** 10.1007/s00256-020-03526-5

**Published:** 2020-06-30

**Authors:** Sevtap Tugce Ulas, Kay Geert Hermann, Marcus R. Makowski, Robert Biesen, Fabian Proft, Ralph Schilling, Torsten Diekhoff

**Affiliations:** 1 Department of Radiology, Charité – Universitätsmedizin Berlin, Campus Mitte, Humboldt – Universität zu Berlin, Freie Universität Berlin, Berlin, Germany; 2 Department of Rheumatology, Charité – Universitätsmedizin Berlin, Campus Mitte, Humboldt – Universität zu Berlin, Freie Universität Berlin, Berlin, Germany; 3 Department of Rheumatology, Charité – Universitätsmedizin Berlin, Campus Benjamin Franklin, Humboldt – Universität zu Berlin, Freie Universität Berlin, Berlin, Germany; 4 Institute of Biometry and Clinical Epidemiology, Charité – Universitätsmedizin Berlin, Campus Mitte, Humboldt – Universität zu Berlin, Freie Universität Berlin, Berlin, Germany

**Keywords:** Computed tomography, Magnetic resonance imaging, Perfusion imaging, Arthritis, rheumatoid, Synovitis

## Abstract

**Objective:**

To evaluate the performance of dynamic contrast-enhanced CT (DCE-CT) in detecting and quantitatively assessing perfusion parameters in patients with arthritis of the hand compared with dynamic contrast-enhanced MRI (DCE-MRI) as a standard of reference.

**Materials and methods:**

In this IRB-approved randomized prospective single-centre study, 36 consecutive patients with suspected rheumatoid arthritis underwent DCE-CT (320-row, tube voltage 80 kVp, tube current 8.25 mAs) and DCE-MRI (1.5 T) of the hand. Perfusion maps were calculated separately for mean transit time (MTT), time to peak (TTP), relative blood volume (rBV), and relative blood flow (rBF) using four different decomposition techniques. Region of interest (ROI) analysis was performed in metacarpophalangeal joints II–V and in the wrist. Pairs of perfusion parameters in DCE-CT and DCE-MRI were compared using a two-tailed t test for paired samples and interpreted for effect size (Cohen’s d). According to the Rheumatoid Arthritis Magnetic Resonance Imaging Score (RAMRIS) scoring results, differentiation of synovitis-positive and synovitis-negative joints with both modalities was assessed with the independent t test.

**Results:**

The two modalities yielded similar perfusion parameters. Identified differences had small effects (d 0.01–0.4). DCE-CT additionally differentiates inflamed and noninflamed joints based on rBF and rBV but tends to underestimate these parameters in severe inflammation. The total dose-length product (DLP) was 48 mGy*cm with an estimated effective dose of 0.038 mSv.

**Conclusion:**

DCE-CT is a promising imaging technique in arthritis. In patients with a contraindication to MRI or when MRI is not available, DCE-CT is a suitable alternative to detect and assess arthritis.

## Introduction

The early detection of inflammatory changes such as synovitis is crucial to prevent the development of structural changes and bone destruction such as erosions [1]. Qualitative evaluation of inflammatory activity is performed using the American College of Rheumatology/European League Against Rheumatism collaborative initiative (ACR/EULAR) criteria [2]. Dynamic contrast-enhanced imaging techniques enable the assessment of synovial enhancement over time in patients with rheumatoid arthritis [3–5]. As a result, a comprehensive evaluation of inflammatory activity in rheumatic joint diseases is possible [6–8].

With dynamic contrast-enhanced magnetic resonance imaging (DCE-MRI), it has become possible to evaluate synovitis and disease activity with sufficient accuracy [9] to quantify the response to therapy in research studies. Thus far, dynamic contrast-enhanced imaging has not been used widely to assess patients with arthritis in clinical practice. One reason is that sophisticated imaging technology and special software tools are required to derive and assess perfusion parameters. Ultrasonography can be offered as an alternative imaging test [10], but the results are often dependent on the experience of the examiner and difficult to quantify [11]. The first studies investigated the capability of computed tomography (CT) in the detection of active inflammation [12, 13]. Dynamic contrast-enhanced computed tomography (DCE-CT) has the potential to provide a differentiated estimation of inflammatory joint lesions through the assessment of perfusion parameters. Furthermore, it offers the superior detection of structural changes intrinsic to CT [14–16]. The use of DCE-CT in clinical practice has become feasible due to dose reduction with low-kVp scanning protocols and the introduction of iterative reconstruction algorithms in recent years [17, 18].

The purpose of our study was to evaluate the potential of low-dose DCE-CT in the detection and evaluation of synovitis of the hand in patients with acute arthritis using DCE-MRI as the standard of reference. CT offers a sensitive alternative to MRI with lower costs, wider availability, shorter examination duration, and superior detection of erosions at the cost of radiation exposure.

## Materials and methods

### Subjects

In our randomized prospective single-centre study, we consecutively investigated 37 patients (according to a calculation of the necessary sample size) with suspected or proven diagnosis of rheumatoid arthritis (RA) and fulfilled the classification criteria of the American College of Rheumatology (ACR) [19]. The patients presented to the rheumatology inpatient or outpatient clinic of our institution from September 2016 to October 2017. Due to the request of the local ethics committee, all patients had to be over 50 years old. Exclusion criteria were contraindications to MRI, e.g. pacemaker or cochlear implants, known allergic reactions to contrast agent as well as patients with reduced kidney function (glomerular filtration rate (GFR) < 60 ml/min/1.73 m^2^) and hyperthyroidism. The final diagnosis was made by board-certified rheumatologists based on clinical (patient’s history, number of swollen and tender joints) and laboratory findings (C-reactive protein, rheumatoid factors (RF) and anti-citrullinated protein antibodies (ACPA)) as well as imaging findings of our study.

The study was approved by the local ethics committee (EA1/259/15) and the Federal Office for Radiation Protection (Z 5–22,462/2–2016-008). All patients gave their written informed consent.

### DCE-CT and DCE-MRI procedures

All patients underwent DCE-CT and 1.5 T DCE-MRI of the clinically dominant hand and wrist. Both scans were performed on the same day with a maximum time interval of 1 h between them. Patients were randomized to DCE-CT first or DCE-MRI first.

Patients were scanned in a prone position with the hand stretched above the head into the scanner and positioning the palm on the table (so-called superman position). The arm was fixed with a sandbag to reduce motion. Contrast agent was administered into the contralateral arm at a flow of 2 ml/s using an intravenous needle (Braunüle®, 1.10 × 33 mm, G20, Vasofix®, B. Braun®; Melsungen, Germany) and an automatic injection device.

### Dynamic contrast-enhanced CT acquisition protocol

DCE-CT was performed on a 320-row CT scanner (Canon Aquilion ONE Vision, Canon Medical Systems; Otawara, Japan) in volume mode with 16 cm z-axis coverage without table movement. We used a tube voltage of 80 kVp and a tube current of 30 mA with a rotation time of 0.275 s. A total of 10 imaging volumes were acquired*.* The scans were obtained 0, 10, 20, 30, 40, 50, 60, 90, 120, and 180 s after contrast injection. We administered a body-weight-adapted dose of 1 ml/kg iopromide (Ultravist 370®, Bayer®; Leverkusen, Germany) directly followed by a bolus of 30 ml sodium chloride. Images were reconstructed with a slice thickness of 0.5 mm using a medium soft tissue kernel without beam hardening compensation.

### Dynamic contrast-enhanced MRI acquisition protocol

DCE-MRI was performed on a 1.5-T Siemens Magnetom Avanto imager (Siemens Healthcare; Erlangen, Germany) using a small flexible 4-channel coil (Siemens healthcare; Erlangen, Germany) for the hand.

Before contrast agent injection, we acquired the following sequences: T1-weighted spin-echo sequences in coronal planes and coronal T1-weighted turbo-inversion recovery-magnitude sequences (TIRM) (for details see Table 1).

**Table 1:** DCE-MRI protocol

	FOV Read [mm]	FOV Phase [%]	Slice thickness [mm]	Number of Slices	TR [msec]	TE [msec]	Resolution Matrix	Flip Angle [degree]
T1	160	100	3	12	401	21	512 × 512	90
TIRM	160	100	3	16	5000	69	256 × 241	150
dVIBE	180	75	3	20	6.5	2.53	192 × 128	17
T1 fs								
Coronal	200	100	3	14	719	11	512 × 256	150
Axial wrist	140	100	4	14	591	15	320 × 192	90
Axial MCP	140	100	4	12	507	15	320 × 192	90

*FOV* field of view, *TR* repetition time, *TE* echo time, *TIRM* turbo-inversion recovery-magnitude, *SWI* susceptibility-weighted imaging, *VIBE* volumetric interpolated breath-hold examination, *T1* T1-weighted imaging, *T1 fs* fat-saturated contrast-enhanced T1 sequence

For perfusion analysis, we acquired coronal fat-saturated dynamic T1-weighted fast 3D gradient-echo volumetric interpolated breath-hold examination (dVIBE) sequences after contrast medium injection. VIBE sequences were obtained every 10 s over 3 min. Postcontrast images were acquired with fat-saturated T1-weighted spin-echo sequences in coronal and transverse slice orientations.

Contrast medium was diluted in isotonic sodium chloride solution at a ratio of 1:4, and an amount of 1 ml/kg was injected. This resulted in a total contrast agent volume of 0.2 ml/kg body weight of gadolinium-DOTA (Dotarem®; Guerbet, France) and sodium chloride volume of 0.8 ml/kg body weight. Thereafter, a bolus of 30-ml pure sodium chloride solution was administered.

### Image reading

Prior to quantitative perfusion analysis, three independent readers (K.G.H. with 18 years, T.D. with 9 years, S.T.U. with 1 year of experience in musculoskeletal imaging) blinded to identifying information, clinical data, and other imaging findings scored the fat-saturated contrast-enhanced T1-weighted images for synovitis according to the Rheumatoid Arthritis Magnetic Resonance Imaging Score (RAMRIS) criteria, defined by the Outcome Measurement in Rheumatology (OMERACT) MRI group [20] to dichotomize the joints in inflamed (RAMRIS synovitis ≥ 1) versus noninflamed (RAMRIS synovitis = 0). The agreement of at least two readers was determined as a reference.

### Image preprocessing

All images were pseudonymized with regard to name, age, and sex. Preprocessing for perfusion maps in DCE-MRI and DCE-CT was performed using an Olea sphere (Version 3.0, Olea Medical; La Ciotat, France). It was performed separately using four different decomposition methods: delay-sensitive and non-adaptive standard singular value decomposition (sSVD), delay-insensitive and non-adaptive circular singular value decomposition (cSVD), delay-insensitive and semi-adaptive oscillation index singular value decomposition (oSVD), and delay-insensitive and adaptive Bayesian decomposition. The singular value decomposition algorithms (SVD) have integrated an upstream threshold for the signal variance to reduce the effects of non-physiological oscillations [21]. The Bayesian technique enables a rigorous probabilistic estimation of haemodynamic parameters based on Bayes’ theorem [22]. In validated simulations, the Bayesian decomposition technique outperforms other techniques in terms of accuracy and robustness against noise [23].

**Fig. 1:**
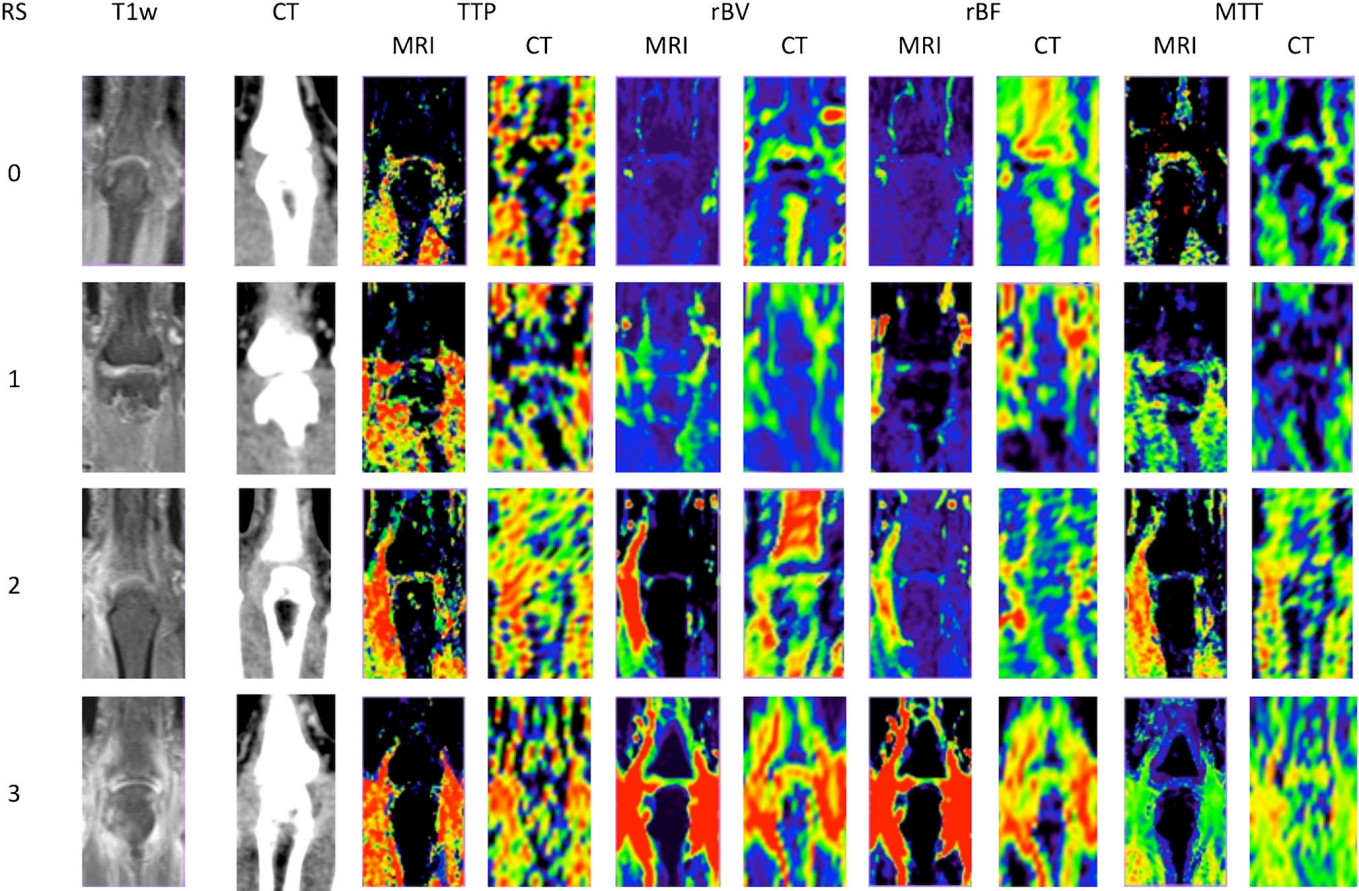
Coronal perfusion maps of metacarpophalangeal joint III for different RAMRIS synovitis scores using standard singular value decomposition in four different patients. RS = RAMRIS synovitis score, MRI = magnetic resonance imaging, CT = computed tomography, TTP = time to peak, rBV = relative blood volume, rBF = relative blood flow, and MTT = mean transit time. The first column shows T1-weighted fat-saturated post-contrast image (T1w) and the second column shows contrast-enhanced CT (CT) images. Perfusion parameters obtained with both modalities increase with RAMRIS scores. For RAMRIS scores 2 and 3, DCE-CT tends to underestimate the severity of synovitis compared to DCE-MRI

Motion correction was applied automatically. To compare perfusion parameters derived from DCE-CT and DCE-MRI, the same ten time points were chosen to generate perfusion maps: 0, 10, 20, 30, 40, 50, 60, 90, 120, and 180 s after contrast agent administration. To calculate the arterial input function (AIF), a region of interest (ROI) was placed manually in the radial artery. The venous output function (VOF) was determined in an ROI manually placed in the vein of the back of the hand. The baseline was defined as the time point at which increasing signal intensity was detectable. Colour-coded perfusion maps with 3-mm slice thickness were calculated for the following parameters: time to peak (TTP), which describes the time to the peak of contrast uptake in the selected ROI, relative blood volume (rBV), relative blood flow (rBF), and mean transit time (MTT). Joints with severe synovitis show increased perfusion parameters, indicated by red colour in the perfusion maps, while blue and black indicate regions with lower perfusion parameters (see Fig. 1).

### Image post-processing and regions of interest

ROI analysis of the computed perfusion maps was performed using Osirix (Version 7.0, Pixmeo SARL; Bernex, Switzerland). Image post-processing was conducted in a coronal orientation. According to the established RAMRIS criteria [20], the ROIs were placed in MCP II, MCP III, MCP IV, MCP V, radioulnar, radiocarpal, and intercarpal joints. ROIs were placed in the region with the highest contrast uptake in inflamed joints. In noninflamed joints, ROIs were placed on the radial side of the joints and on the radial mediocarpal joints for the intercarpal region. All ROIs were circular, and an area of 10 mm^2^ was used for evaluation. The same ROI positioning was used for all four decomposition techniques and the two imaging modalities. The mean value of each measurement in each ROI was noted.

### Radiation exposure

The radiation exposure (estimated effective dose) of DCE-CT examinations was calculated using the overall dose-length product (DLP) and a conversion coefficient of 0.0008 [mSv × mGy^−1^ × cm^−1^].

### Statistical analysis

Statistical analysis was performed using R (RStudio, Version 1.1.463). The results of ROI analysis were collected and summarized for the respective perfusion parameters. The mean and standard deviation were calculated for each perfusion parameter and decomposition technique separately. Negative values of ROI analysis due to artefacts were excluded from statistical analysis. To identify significant differences between perfusion parameter pairs in DCE-CT and DCE-MRI, we performed a two-tailed paired t test. The results of the paired t test were interpreted based on the effect size using Cohen’s d with the following classification: no effect < 0.1, small 0.2–0.4, medium 0.5–0.7, large 0.8–≥ 1.0 [24]. Furthermore, means, standard deviations, and 95% confidence intervals were used for interpretation of differences. In a second step, an independent two-tailed t test was used to investigate whether DCE-CT is able to differentiate between inflamed and noninflamed joints with a performance similar to that of DCE-MRI (according to scoring results). A *p* value < 0.05 was considered statistically significant.

For assessment of agreement between DCE-CT and DCE-MRI, Bland-Altman plots of TTP, rBV, rBF, and MTT were created for inflamed joints separately for each of the four decomposition techniques.

## Results

### Subjects and image reading

**Fig. 2:**
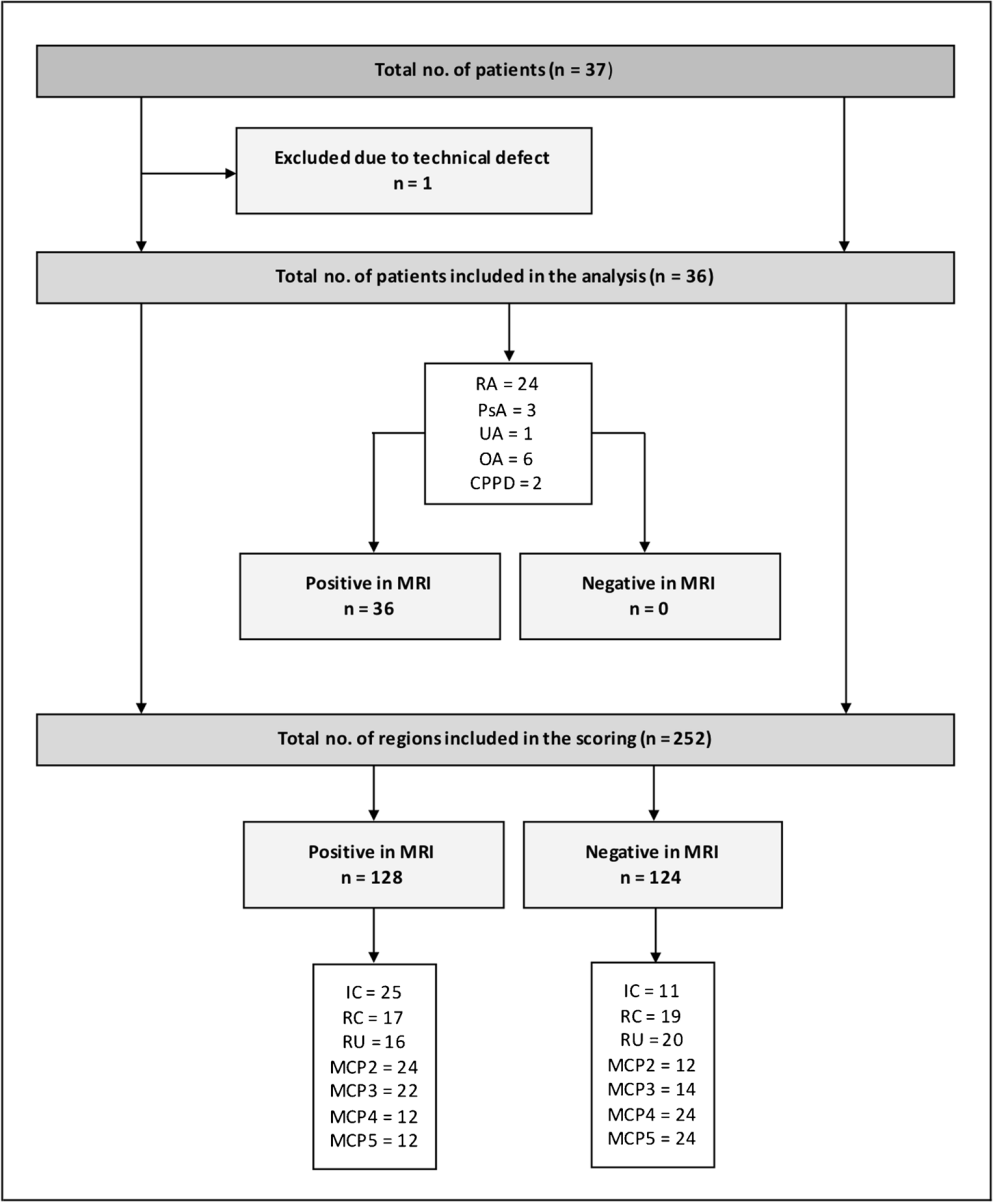
Flowchart of study inclusion and results of RAMRIS scoring for synovitis. RA = rheumatoid arthritis, PsA = psoriatic arthritis, UA = undifferentiated arthritis, OA = osteoarthritis, CPPD = calcium pyrophosphate deposition disease, IC = intercarpal joint, RC = radiocarpal joint, RU = radioulnar joint, and MCP2–5 = metacarpophalangeal joints 2–5

The study included 37 patients (27 women; mean age 60.1, 50–77 years) with a mean weight of 77.3 kg (SD 14.3), a mean C-reactive protein (CRP) of 18 mg/dl (SD 42.6), and a mean duration of symptoms of 3.9 years (SD 4.84). In one patient, DCE-MRI could not be performed due to technical problems during the examination and was therefore excluded from further statistical analysis. The study population and RAMRIS scores are presented in Table 2 and Fig. 2. ACPA was positive in 12 patients (33.3%), while this was the case for RF in 8 patients (22.25%). Fourteen patients were never treated, three were treated with NSAIDs, 6 received only corticosteroids, 7 received disease-modifying antirheumatic drugs (DMARDs), and 6 received biologicals. No patient received a corticosteroid injection prior to the examination.

**Table 2:** Characteristics of the study population

Characteristics	*Total*	*RA*	*Non-RA*
No. of participants	36	24	12
Women	26	17	9
Men	10	7	3
Age (y)	60.4 ± 7.1 (50–77)	60.4 ± 7.2 (50–77)	58.6 ± 5.6 (52–68)
Women	59.2 ± 6.2 (50–74)	58.8 ± 6.6 (50–74)	60.0 ± 5.7 (52–68)
Men	63.4 ± 8.6 (52–77)	67.3 ± 6.9 (56–77)	54.3 ± 3.2 (52–58)
Weight [kg]	77.6 ± 14.4	79.7 ± 15.3	73.8 ± 12.0
Symptom duration (y)	4.2 ± 4.9	3.8 ± 4.7	5.0 ± 5.4
CRP [mg/l]	15.9 ± 40.3	21.9 ± 47.0	1.7 ± 1.6
No. of ACPA-positive	12	11	1
No. of RF-positive	8	8	0

The values are given as follows: means ± standard deviation and range in parentheses. *RA* rheumatoid arthritis, *CRP* C-reactive protein, *ACPA* anti-citrullinated protein antibodies, *RF* rheumatoid factors, ACPA-positive was defined as ACPA > 20 U/ml and RF-positive as RF > 14 U/ml)

Twenty-one patients underwent DCE-CT before DCE-MRI. According to RAMRIS scores, 50.8% (128/252) of the regions analysed were positive for synovitis (see Fig. 2).

### Image preprocessing

**Fig. 3:**
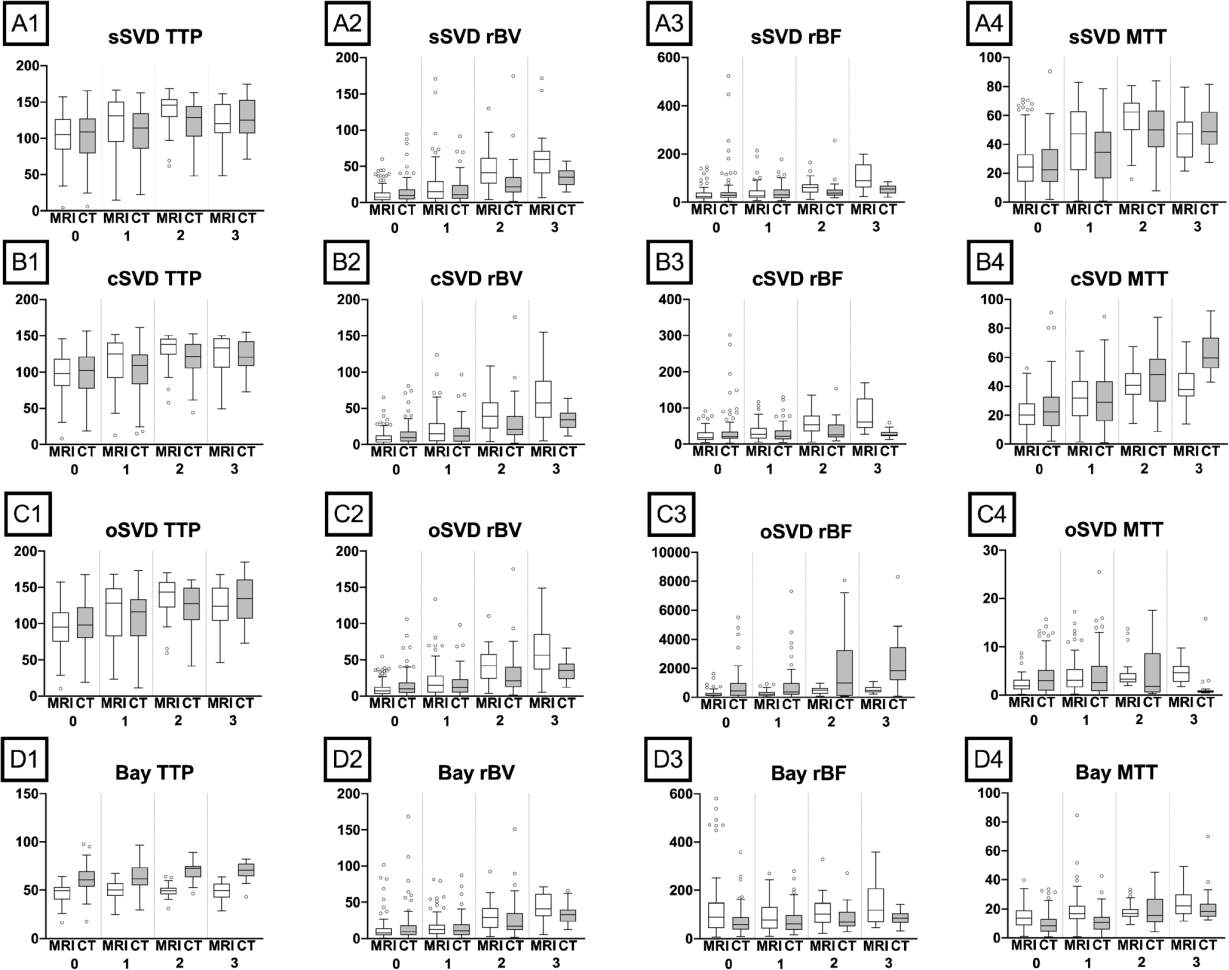
Boxplots of perfusion parameters versus RAMRIS grading for the four decomposition techniques investigated. sSVD = standard singular value decomposition, cSVD = circular singular value decomposition, oSVD = oscillar singular value decomposition, Bay = Bayesian decomposition. TTP = time to peak, rBV = relative blood volume, rBF = relative blood flow, and MTT = mean transit time. Y axis: perfusion parameter. X axis: RAMRIS synovitis score. Overall, perfusion parameters determined by DCE-CT and DCE-MRI increase with the RAMRIS synovitis score. However, for most parameters, DCE-CT seems to be less responsive than DCE-MRI

In all patients, perfusion maps of TTP, rBV, rBF, and MTT were computed separately for preprocessing with sSVD, cSVD, oSVD, and the Bayesian algorithm. All data were accepted and successfully post-processed by the software. The post-processing and quantitative measurements took approximately 10 min per patient, modality, and reconstruction algorithm. These time, investments add up to approximately 48 h of continuous work for the whole study population. Examples of perfusion maps are shown in Fig. 1. In Fig. 3, boxplots of perfusion parameters of the different RAMRIS gradings are presented.

### Image post-processing and regions of interest

Means, standard deviations, t test results, and effect sizes as well as differences in means and standard deviations of differences and 95% confidence intervals of DCE-CT and DCE-MRI for TTP, rBV, rBF, and MTT using the different decomposition techniques are presented in Table 3. Overall, our results revealed no significant differences between perfusion parameters determined with the two modalities, particularly for rBF in sSVD and TTP in oSVD. The observed differences were of small effect (d = 0.01–0.4). The results indicate that DCE-CT is able to distinguish between inflamed and noninflamed joints (Table 4).

**Table 3:** Results of the paired two-tailed t-test

Decomp.	Param.	MRI-derived mean	MRI-derived SD	CT-derived mean	CT-derived SD	Mean of diff.	SD of diff.	95% CI of diff.	p value	Effect size (Cohen’s d)
sSVD	TTP	116.2	32.7	110.1	32.4	6.1	40.6	0.9 to 11.2	0.019	0.2
	rBV	24.5	29.1	19.1	20.1	5.4	25.3	2.2 to 8.5	0.001	0.2
	rBF	43.7	38.9	44.5	53.4	− 0.7	60.0	− 8.2 to 6.8	0.845	0.01
	MTT	37.5	22.0	34.0	19.9	3.5	23.6	0.5 to 6.5	0.020	0.2
cSVD	TTP	110.3	30.4	103.6	32.4	6.7	38.4	1.9 to 11.5	0.007	0.2
	rBV	22.4	26.0	18.8	19.9	3.6	23.3	0.7 to 6.5	0.015	0.2
	rBF	36.4	30.9	32.6	34.8	3.8	43.9	− 1.7 to 9.2	0.176	0.09
	MTT	28.8	15.0	32.3	20.5	− 3.5	19.2	− 5.9 to − 1.1	0.004	0.2
oSVD	TTP	109.8	36.1	108.6	33.8	1.2	42.5	− 4.0 to 6.5	0.644	0.03
	rBV	22.7	25.6	19.6	20.9	3.1	23.9	0.1 to 6.1	0.044	0.1
	rBF	307.3	248.2	1066.6	1480.8	− 759.2	1387.6	− 932.4 to − 586.0	< 0.001	0.5
	MTT	3.3	2.7	4.0	4.3	− 0.6	5.1	− 1.3 to 0.0005	0.050	0.1
Bay	TTP	48.7	8.9	63.6	12.7	− 14.9	13.5	− 16.6 to − 13.2	< 0.001	1.1
	rBV	20.3	25.9	23.2	50.3	− 2.9	53.9	− 9.6 to 3.9	0.401	0.05
	rBF	112.7	92.3	146.3	452.8	− 33.6	459.3	− 90.8 to 23.6	0.248	0.07
	MTT	17.6	11.9	12.4	9.2	5.2	13.9	3.4 to 6.9	< 0.001	0.4

*Decomp*. decomposition technique, *Param.* perfusion parameter, *SD* standard deviation, *diff.* difference, *95% CI* 95% confidence interval of the difference, *sSVD* standard singular value decomposition, *cSVD* circular singular value decomposition, *oSVD* oscillar singular value decomposition, *Bay* Bayesian decomposition

**Table 4:** Results of the independent t-test

Decomp.	Param.	+MRI mean ± SD	-MRI mean ± SD	MRI-derived p value	+CT mean ± SD	-CT mean ± SD	CT-derived p value
**sSVD**	TTP	126.2 ± 32.0	105.5 ± 29.9	<0.001	116.5 ± 31.7	103.4 ± 31.8	0.001
	rBV	36.9 ± 34.5	10.9 ± 11.6	<0.001	23.7 ± 22.3	14.2 ± 16.1	<0.001
	rBF	55.4 ± 45.0	31.0 ± 26.0	<0.001	44.6 ± 33.3	44.4 ± 68.8	0.982
	MTT	47.4 ± 21.1	26.6 ± 17.4	<0.001	40.7 ± 20.4	26.7 ± 16.5	<0.001
**cSVD**	TTP	120.9 ± 29.4	99.2 ± 27.4	<0.001	109.5 ± 31.6	97.4 ± 32.1	0.003
	rBV	34.2 ± 30.5	9.8 ± 10.2	<0.001	23.7 ± 23.1	13.7 ± 14.2	<0.001
	rBF	48.5 ± 36.1	23.6 ± 16.9	<0.001	31.2 ± 24.1	34.1 ± 43.4	0.522
	MTT	35.9 ± 14.9	21.3 ± 11.1	<0.001	40.2 ± 21.4	24.0 ± 15.8	<0.001
**oSVD**	TTP	123.0 ± 35.6	96.0 ± 31.3	<0.001	116.5 ± 34.2	100.3 ± 31.5	<0.001
	rBV	34.3 ± 29.9	10.3 ± 10.5	<0.001	24.5 ± 23.6	14.4 ± 16.2	<0.001
	rBF	372.9 ± 254.1	246.5 ± 239.0	<0.001	1397.8 ± 1809.4	716.1 ± 908.8	<0.001
	MTT	4.3 ± 3.2	2.3 ± 1.4	<0.001	3.9 ± 4.9	4.0 ± 3.7	0.960
**Bay**	TTP	49.7 ± 8.7	47.7 ± 9.0	0.065	66.0 ± 12.4	61.1 ± 12.6	0.003
	rBV	27.7 ± 30.9	12.6 ± 16.0	<0.001	22.5 ± 21.5	24.0 ± 68.8	0.818
	rBF	107.1 ± 70.3	119.2 ± 110.0	0.296	96.5 ± 185.7	198.7 ± 616.7	0.074
	MTT	20.5 ± 14.1	14.7 ± 7.8	<0.001	14.9 ± 10.2	9.9 ± 7.2	<0.001

*Decomp*. decomposition technique, *Param.* perfusion parameter, *+MR/+CT* results for inflamed joints in MRI und CT, *−MRI/-CT* results for noninflamed joints, *SD* standard deviation, *sSVD* standard singular value decomposition, *cSVD* circular singular value decomposition, *oSVD* oscillar singular value decomposition, *Bay* Bayesian decomposition

**Fig 4:**
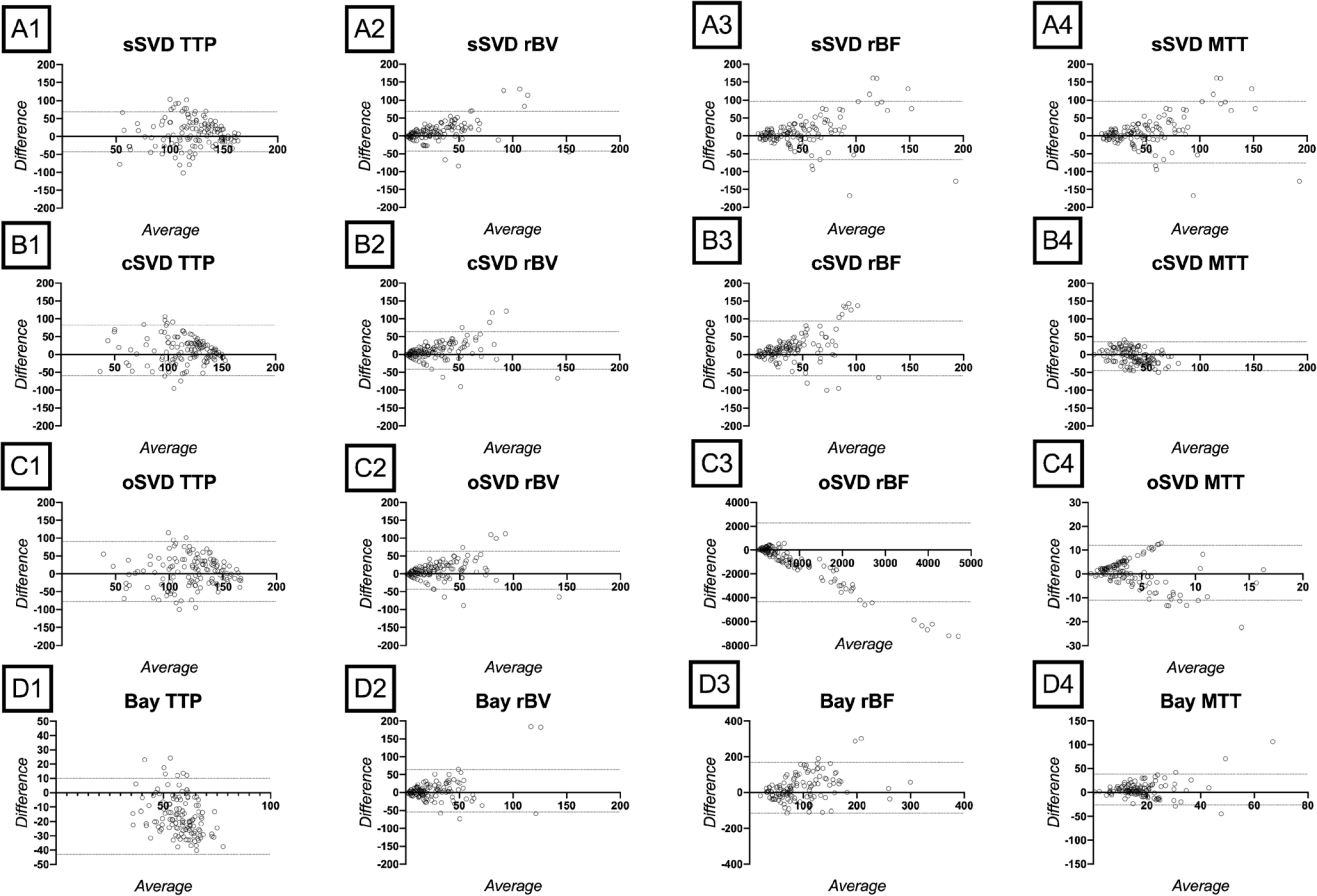
Bland-Altman plots. sSVD = standard singular value decomposition, cSVD = circular singular value decomposition, oSVD = oscillar singular value decomposition, Bay = Bayesian decomposition. TTP = time to peak, rBV = relative blood volume, rBF = relative blood flow, and MTT = mean transit time. Y axis: difference between DCE-MRI and DCE-CT. X axis: average DCE-MRI and DCE-CT. There is good overall agreement between the two modalities, but DCE-CT tends to underestimate disease activity in joints with severe inflammation

Figure 4 presents Bland-Altman plots for TTP, rBV, rBF, and MTT in regions with synovitis for each of the four decomposition techniques, namely, sSVD, oSVD, cSVD, and Bayesian technique. The plots show that joints with severe inflammation tended to be underestimated in DCE-CT.

### Radiation exposure

The total DLP was 48 mGy*cm, resulting in an estimated effective dose of 0.038 mSv.

## Discussion

This is the first study investigating the innovative technique of low-dose DCE-CT for the assessment of synovitis of the hand in patients with active arthritis using four different decomposition techniques. We show that DCE-CT is able to differentiate between inflamed and noninflamed joints and between the different grades of the semi-quantitative RAMRIS synovitis scores with no major differences between the four decomposition techniques studied. While DCE-CT tends to underestimate severe inflammation, it provides similar perfusion parameters as DCE-MRI and is able to identify joints with synovitis. In this regard, rBF shows the best agreement between the two modalities. Differences in perfusion parameters identified between the two modalities have small effects.

DCE-CT is more sensitive than MRI or traditional radiography in detecting soft tissue calcification and smaller erosions. Thus, CT allows a broader differential diagnosis. Due to the upcoming ultra-low-dose protocols [25], CT has the potential to be applied in routine care. DCE-CT also allows automated image post-processing and reading. Furthermore, it also offers a gadolinium-free alternative, as MRI contrast media can result in gadolinium deposition with as yet unclear effects [26, 27]. Additionally, CT is faster and more readily available. In our previous study, we showed that patients prefer CT than MRI and feel more comfortable in a CT scanner [13]. For comparability with MRI, CT examinations in our study were performed in the superman position as well. In the clinical setting, CT examinations can be performed with the patient sitting comfortably next to the gantry.

Sonography has several advantages in the detection of synovial inflammation using power-Doppler with a high diagnostic ability in depicting erosions, synovial hypertrophy, and hyperaemia [28]. However, its diagnostic performance varies with the examiner’s experience [11], and the quantification of inflammation and structural lesions for follow-up is limited [29]. For this reason, a comparison of sonography with DCE-CT and DCE-MRI is unfeasible. Modern CT techniques allow the depiction of bone marrow oedema [30]. DCE and dual-energy CT might help to detect osteitis, and the combined use of both techniques may provide further benefits.

Our study was specifically planned to compare DCE-CT and DCE-MRI. We used adapted doses of contrast agent in DCE-CT and DCE-MRI to create comparable conditions. The same software was used to generate perfusion maps in DCE-CT and DCE-MRI and for quantitative ROI analysis. However, our study has some limitations. The different inherent properties of the imaging modalities and contrast agents must be taken into account [31]. We investigated only 36 patients, adhering to our estimate of the necessary sample size. We are aware that, despite showing the absence of significant differences in perfusion parameter means between CT and MRI, our study is not designed to establish equivalence or non-inferiority. Gadolinium retention in patients who underwent MRI first might have influenced the CT enhancement. Using a low-dose CT protocol can both limit the ability to detect subtle bone changes and lead to measurement artefacts that can be minimized by applying higher radiation to improve the contrast-to-noise ratio. According to the new EULAR recommendation, MRI is no longer used for the early diagnosis of RA because of its low specificity [32]. Further studies investigating DCE-CT using an optimized image reconstruction protocol (e.g. artificial intelligence) in larger numbers of patients are needed. In addition, a comparison with 3 T scanners would be of particular interest.

DCE-CT has the potential to assess synovitis. Using four decomposition techniques, we showed that there is overall good agreement between DCE-CT and DCE-MRI, while DCE-CT tends to underestimate some perfusion parameters. In patients with a contraindication to MRI or when MRI is not available, DCE-CT is a suitable alternative to detect and assess arthritis.

In conclusion, low-dose DCE-CT can differentiate inflamed and noninflamed joints in patients with suspected RA. Additionally, DCE-CT produces perfusion parameters comparable with DCE-MRI.
